# Can a programme to support parents reduce both violence against women and violence against children? A pre-post pilot test in Zimbabwe

**DOI:** 10.3389/fgwh.2026.1759345

**Published:** 2026-07-06

**Authors:** Natalie Davidson, Catherine L. Ward

**Affiliations:** Department of Psychology and Safety and Violence Initiative, University of Cape Town, Cape Town, South Africa

**Keywords:** parenting, parenting for lifelong health, parenting programmes, prevention, violence against children, violence against women

## Abstract

**Introduction:**Violence against women (VAW) and violence against children (VAC) interconnect in a variety of important ways. Parenting programmes may offer promise for preventing both forms of violence, through supporting co-parenting as an avenue for reducing inter-parental conflict, and for engaging men without stigma.

**Method:** This paper presents results from a pilot study with 50 families of the new Co-parenting Sinovuyo Caring Families Programme for Parents and Teens, which took place in rural Manicaland, Zimbabwe. It is a version of the Parenting for Lifelong Health (PLH) Teen programme, adapted to include a more explicit focus on preventing VAW and the engagement of fathers for the Zimbabwean context. A mixed methods embedded intervention design was used to assess changes in VAW and VAC from baseline to endline. Qualitative data included discussions with participant advisory boards before the pilot, individual interviews with participants at the endline, and a focus group discussion with all parenting group facilitators at the endline. Quantitative data included assessments at the endline and baseline with the primary outcome of intimate partner violence (IPV).

**Results:** This programme led to significant reductions in reported physical IPV, co-parenting struggles, harsh parenting, parenting stress, and teen behaviour problems, but not in emotional IPV, teen-reported IPV, or attitudes to harsh punishment of children. There were significant increases in positive parenting and equitable gender attitudes. These quantitative findings are supported by the qualitative findings with participants and facilitators highlighting improved relationships between themselves and their community, families doing things together and less conflict in the home.

**Conclusion:** This programme is promising for the prevention of VAW and VAC simultaneously.

## Introduction

Violence against women (VAW) and violence against children (VAC) are serious, prevalent, and global public health problems (1, 2). Rates tend to be higher in low- and middle-income countries (LMICs) (3–6) and Zimbabwe is no exception: the 2019 Multiple Indicator Cluster Survey (MICS) found that 40.8% of children age 1–14 had experienced some form of physical punishment, and 39.4% of women aged 15-49 had experienced physical violence, with 71.6% reporting that the perpetrator was their current partner/husband (7, 8). The most likely reality in most homes where violence occurs, is that these two problems co-occur (9–12).

These two forms of violence share many of the same consequences. Along with the devastating effects of child maltreatment on children's mental and physical health (13–16), VAW in the home can have an equally and similarly detrimental effect on children in the households (17, 18). It can hamper children's development (19), be a risk factor for mental health difficulties (14, 20), and lead to physical injury as collateral damage (21). Consequences for women experiencing VAW may include serious physical injuries, mental health issues (22) and problems with parenting (23, 24). This violence has been shown to affect the parenting potential of caregivers (23). It is associated with less maternal engagement and more use of corporal punishment (19, 25). While some mothers may struggle to engage with their children when experiencing abuse, others may turn to authoritarian parenting to stop any children's behaviour that could aggravate their abuser (19).

The co-occurrence of VAW and VAC likely arises because they share numerous risk factors (26). These risk factors include lower education levels, higher levels of mental distress, lower socio-economic status and higher levels of alcohol use (4, 27, 28) as well as having experienced abuse as children, having witnessed violence between parents, and poor parent-child relationships (4, 29). The prevalence of past experiences of VAC and VAW in those who are violent in their current family relationships implies that in preventing family violence now, future violence may also be prevented (30). Gender norms characterised by patriarchy are another risk factor for both forms of family violence (26). Norms favouring men's dominance and control that perpetuate and maintain VAW and VAC (27, 31) and norms that see violence as acceptable and familial privacy as a priority, are key drivers of violence within the family (3, 26, 32). While there are few studies on gender norms in Zimbabwe, those that do exist find a recurring norm of a clear, traditionally patriarchal division of labour within the home, where men are seen as breadwinners while women are responsible for parenting and the household (33–35).

Positive co-parenting is negatively correlated with VAW (36) and with VAC and VAW co-occurrence (28, 37). Positive co-parenting requires a good parental relationship with good problem-solving skills, emotion regulation, conflict management and support for one another (38, 39). A recent systematic review found that co-parenting programmes have a small but significant positive effect on parents' well-being, the child's adjustment, the parent-child relationship and the relationship between parents (37), and more gender-equal couple relationships are protective against VAW (40). Together, this suggests that healthy gender-equal couple relationships may be key to preventing both VAW and VAC.

There is robust evidence that parenting programmes are an effective way to prevent child maltreatment in the family (41–43). Parenting programmes have also been identified as promising for additionally reducing adolescent intimate partner violence (IPV) through reducing violence exposure in the home (44, 45). Interventions addressing VAW and VAC are still limited, particularly in Africa, where only a few have been rigorously evaluated. The majority of these are parenting programmes (47%) (46). Further, the conflict resolution and problem-solving strategies of parenting programmes could easily be expanded to address family violence more broadly. Parenting programmes also offer an opportunity to address household roles and thus gender norms. For this reason, the World Health Organisation's (WHO) RESPECT framework for the prevention of VAW (47) and their INSPIRE framework for the prevention of VAC (48) both highlight parenting programmes and norm change as key prevention strategies.

Given the importance of working to promote healthy co-parenting relationships, interventions should include both co-parenting partners to improve their relationship and ensure they work together to enact new, positive parenting behaviours (37). Historically, most parenting programmes have struggled to recruit fathers (49), despite the importance of father engagement for child development: father engagement with children has been found to reduce behaviour problems in boys and mental health problems in girls and improve cognitive development in all children (50). It should be noted that father engagement is linked to positive outcomes when it is characterised by care, warmth and non-violence (51). Men may be reluctant to engage in parenting programmes, at least in part because of the widely-held social norm that parenting is “women's work” (52) and deficit models of fathering (53), which focus on men's insufficiencies as parents (54). Father engagement in parenting programmes is also likely to protect against programme dropout, and to improve co-parenting and programme outcomes (53, 55). A recent study sought to identify the core elements and change mechanisms of effective fatherhood programmes by examining two well-known fatherhood interventions focused on addressing VAW and VAC. They highlight the importance of applying a gender transformative approach that focuses on women's agency, encouraging positive masculinities, and intervening in root causes of intergenerational violence as well as in formative stages of men's lives and nurturing local ownership (56). The involvement of fathers would be crucial for a programme that also intends to reduce VAW, as both parents would need to be present to agree to parenting and partner conflict resolution strategies, and emphasising the importance of co-parenting may provide a stigma-free strategy for engaging men in the programme.

Given the commonalities between VAC and VAW, the importance of prevention, and the potential attractiveness of parenting programmes for engaging men in particular in discussions of caregiving and conflict resolution, it is clear that prevention efforts should be integrated. We therefore took advantage of the success of the Parenting for Lifelong Health Programme for Parents and Teens (57–60) to try to address VAC and VAW together, by adapting the programme to include an emphasis on co-parenting, couples conflict resolution, and gender transformative content; and then testing the adapted programme in a pre-post pilot study.

## Aims and objectives

This study sought to pilot the Parenting for Lifelong Health Programme for Parents and Teens (known locally as the Sinovuyo Caring Families Programme), adapted to include a focus on addressing VAW, and assess its impact on reducing VAW and VAC and provide recommendations for refinement and scalability.

## Method

### Study setting

This study was conducted in the rural area of the Mudzimundirenge Ward of Mutare District, Manicaland province. The Manicaland province is characterised by high levels of poverty and a lack of basic facilities. In the province, 62% do not have electricity in their home, only 58.9% have access to basic drinking services, 22.9% use an open pit latrine and 22.5% are in the poorest quintile of the wealth index (7). Data from the 2022 Population and Housing Census show that around 16,212 people lived in this ward, with a higher female population than male, likely because of migrant labour (61) and an average household size of 3.9 (62).

### Evaluation design

We used mixed methods with an embedded intervention design (63). Both qualitative and quantitative data were collected to establish both the circumstances of the target population as well as the process and outcomes evaluations (64, 65). The Medical Research Council of Zimbabwe, MRCZ/A/2929, The Research Council of Zimbabwe, No. 04704 and the Ethics Committee in the Department of Psychology at UCT, PSY2020-20 approved this study.

### The programme

**Figure 1:**
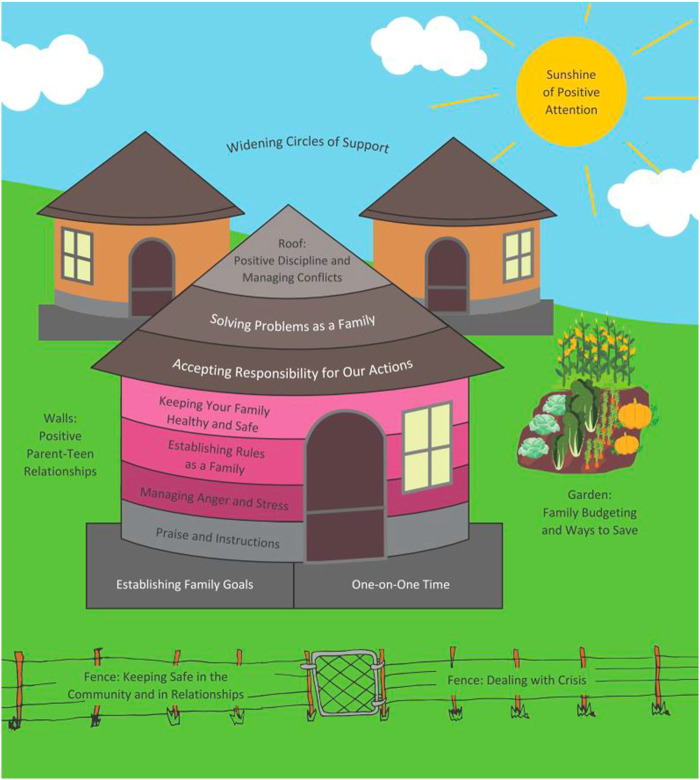
Content of the programme. [Parenting for Lifelong Health. Sinovuyo Caring Families Programme for Parents and Teens: Co-parenting adaptation. Parenting for lifelong health. (2022).] [Creative Commons Attribution-No Derivatives and NonCommercial 4.0 International Public License.] [Parenting for Lifelong Health.]

The Co-Parenting Sinovuyo Caring Families Programme for Parents and Teens programme was a 12-week group-based family strengthening programme for woman caregivers, men caregivers and their children aged 10 to 17. Details about the adaptation can be found in our paper on the formative research for this programme (66) and session content is outlined in Figure 1. As with other PLH programmes, it is designed for resource-poor environments and was implemented by lay community members, who received a 5-day training and subsequent weekly coaching sessions from PLH master trainers in Zimbabwe. Like the original programme, training included the content of the programme as well as skills and techniques to foster positive relationships between the facilitators and participants. Additionally, for this adapted version, the training also included material on IPV and handling disclosure of abuse. These facilitators were provided with a detailed handbook, and participants received simple printed information sheets in six sessions taken from the handbook, which can be accessed at https://doi.org/10.25375/uct.28661624 A key component of this programme is its inclusion of simple mindfulness activities and exercises to promote good mental health and ease any existing symptoms. Programmes should be alert to any symptoms and make timely referrals as necessary. There are 12 weekly sessions. The key changes made to the original programmes were the inclusion of one male co-parent as well as one woman co-parent and one adolescent; three facilitators (including one man); integration of gender-equitable messaging throughout; and opportunities to reflect on the co-parenting relationship.

Sessions lasted around two to three hours and were conducted in community venues such as community halls, churches, and schools. Some sessions were designed to include all three family members, while for others, the male caregivers, women caregivers and teens discussed or did an activity separately. For example, when discussing how to deal with negative emotions the group was split into three groups (men, women and teens) to provide a safe space for discussion. Other activities split the group into their family trios, to give families a chance to discuss things privately and practice new skills before using them at home. The programme's content is interactive and collaborative, including role-plays, games and discussions. Each participant was partnered with a similarly aged, gendered participant, a “Sinovuyo Buddy”. Follow-up home visits were organised to help those who missed sessions or were identified as struggling.

Communities were brought together by the programme facilitators in collaboration with the local ward councillor. Families with adolescent children were specifically invited to these events. Male caregivers were also purposefully invited by emphasising that their engagement was crucial for a family to be able to join the programme. Plan International Zimbabwe implemented this programme for three months between May and July 2023. Each week, facilitators were asked to complete forms about their reflection on the implementation of that week's session, including whether they were unable to complete any of the activities and, if so, the reasons for that, as well as suggestions to improve the session.

### Participant recruitment and sample size

A community meeting that included Plan International staff, the research team, government officials and community representatives, explored who could be nominated to be programme facilitators and research assistants (63–65). Community representatives emphasised that the programme needed to be evenly spread across the ward, and identified four geographic clusters for programme delivery. One family from each cluster was nominated (with an additional family from the biggest cluster) to serve on mother, father, and teen participant advisory boards. These advisory boards were interviewed in focus group discussions to provide feedback on the programme and suggestions for our recruitment strategy. One additional family was nominated from one cluster because it was the largest of the clusters. There were to be three facilitators, one of whom needed to be male, in each group, although one group ended up with four. Our experience with other PLH programmes suggested that 15 families were the maximum group size if parents were to engage fully and facilitators managed the group. On average, there were 12-13 families per session, with at most 39 participants in a session. This large group was managed in two ways: by having three trained facilitators and making sure that most activities and discussions were held in smaller groups or in family trios.

In terms of suggestions for male recruitment and engagement, the participant advisory boards' main suggestion was to have men see that other families were benefiting from the programme. Based on this, the facilitators of the programme recruited the families, emphasising the benefits of the programme. There were no exclusion criteria, but facilitators were briefed to encourage families having conflicts to take part. All inclusion and eligibility criteria were based on a pragmatic approach, and participants were only to be excluded if they had a protection order against them to ensure the safety of the other participants. In practice, no parents were excluded.

Ultimately, 50 families were recruited, with both parents (one male co-parent and one woman co-parent) and one teen participating in the programme. Parents were defined as either biological or non-biological caregivers. After the programme ended, members of the five advisory board families and members of a further five families, were invited, to participate in individual qualitative interviews after the programme ended. This resulted in a total of 30 participants for the qualitative component of the study (10 woman caregivers, 10 male caregivers and 10 teens). The effect sizes of other parenting interventions on parenting practices range from 0.12 to 0.578 (57, 67–69). Based on these sizes, this sample size and a cautiously optimistic estimate of the population effect size of 0.349, the study should have a power of 0.995.

Families who took part in the programme, the facilitators of the programme (*n* = 13) and three implementation staff from Plan International Zimbabwe were all invited to give informed consent to participate in the study.

### Data collection

**Figure 2:**
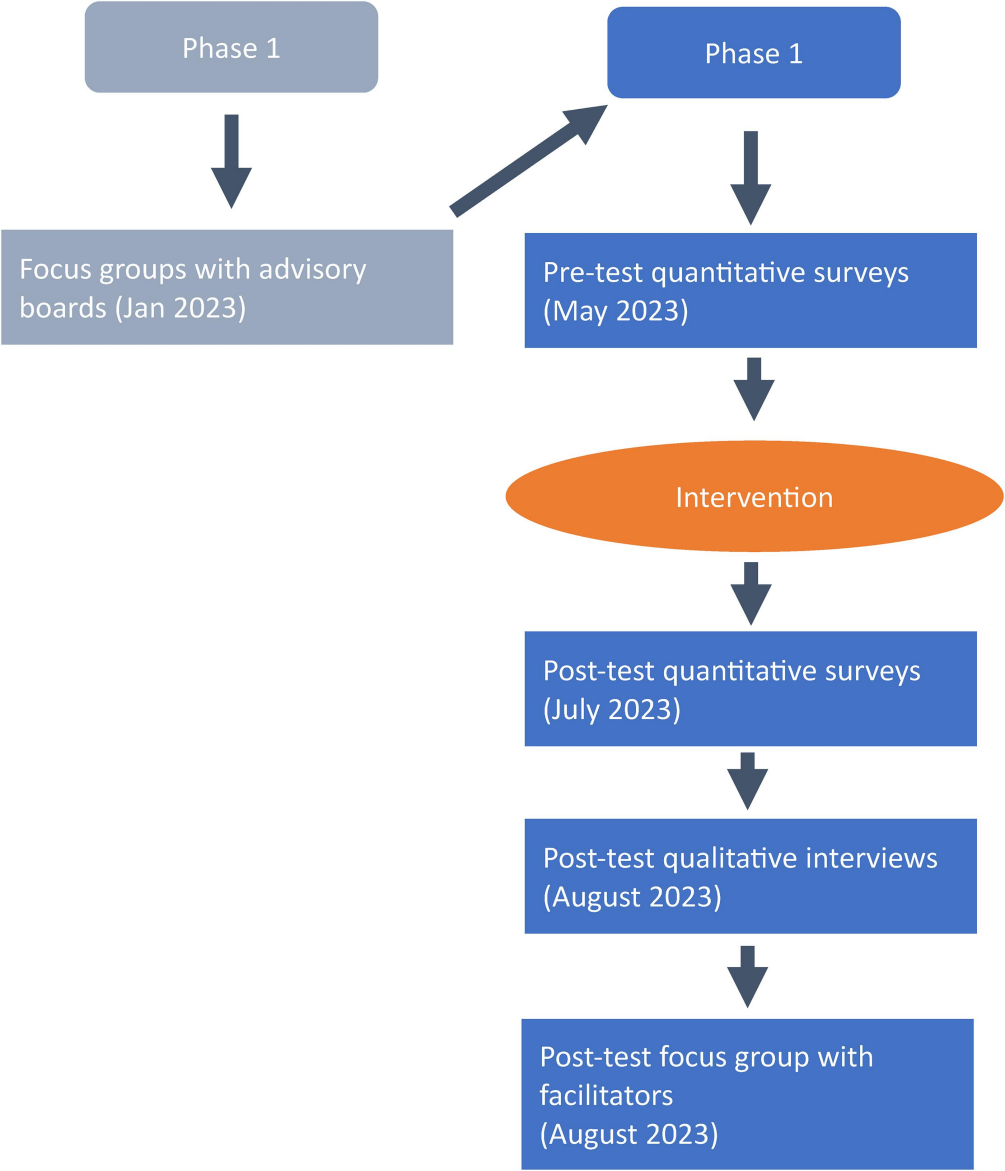
Data collection phases.

There were two phases of data collection (see Figure 2). Firstly, there were hour-long focus group discussions with each of the woman caregiver, male caregiver and teen participant advisory boards prior to implementation, which gave feedback on the adapted programme and how best to recruit other participants (*n* = 5). This was done in January 2023. Secondly, pre- and post-test standardised questionnaires were administered before and after the intervention to assess whether changes had occurred (70). Pre-test data were collected before the start of the intervention (May 2023) and post-test data directly after the intervention (July 2023).

In addition, after the programme had ended, qualitative data was collected in the form of individual interviews with ten male caregivers, ten woman caregivers and ten teen participants, including the five families who participated in the participant advisory board discussions before the programme, along with five other families who participated in the programme in August 2023. The interviews were semi-structured to have some flexibility for the participants to fully share their experiences of the adapted programme but also make sure that the interviews were directly comparable (71). The interviews focused on understanding changes in relationships, including parent-child and co-parenting; the most enjoyed and least enjoyed aspects of the programme; and suggestions for improvement. A focus group discussion with the facilitators after the programme ended focused on the length of the programme, successful and unsuccessful aspects/sessions, differences in participation among different participants and suggestions for improvement.

Participants were interviewed in their homes (after ensuring privacy for the interview) by local Shona-speaking trained research assistants who then transcribed and translated the interviews. Because of political events in Zimbabwe and concerns about fieldwork safety, the focus group discussion (the last piece of data collection) was carried out using Microsoft Teams. All the facilitators were gathered in one room with a local Shona speaker who was able to translate for the interviewer, while the interviewer joined remotely.

### Outcome measures

This study utilised a range of standardised measures. There were three different questionnaires, one for the teens, one for the women caregivers and one for the male caregivers.

#### Primary outcome: IPV

This was assessed using the Revised Conflict Tactics Scales (CTS2S) Short Form (72). The women's questionnaires were the only ones that included questions about intimate partner violence, to prevent men from realising that these questions had been asked, in case this questioning put women in danger (73). The scale has good psychometric properties (72) which have been maintained in studies in various LMIC countries (74), including South Africa (75).

#### Secondary outcomes

##### Parenting practices

The ISPCAN (International Society for Prevention of Child Abuse and Neglect) Child Abuse Screening Tool for Trials (ICAST-Trial) (76) was used to assess abusive parenting with self-report from parents and teens, who reported on both their parents together. Other parenting practices, including poor supervision of teens, erratic discipline, corporal punishment, positive parenting and involved parenting, as reported by adolescents and caregivers, was measured using the Alabama Parenting Questionnaire (77). Attitudes towards corporal and emotional punishment were reported by both parents and teens, using the ICAST-Trial attitudes subscale (76).

Parenting stress was reported by caregivers, using the Parental Stress Scale (78). Caregivers reported on their co-parenting using the exposure to conflict, co-parenting undermining and division of labour subscales of the Co-parenting Relationship Scale (79), which has successfully been used in various LMIC countries (80).

##### Externalising behaviour

Both caregivers and adolescents completed the Child Behaviour Checklist's rule-breaking and aggression subscales (81).

##### Inter-Parental conflict

Conflict between parents was reported on by adolescents using selected items from the Children's Perception of Interparental Conflict Scale (CPICS) (82) and the Child Exposure to Domestic Violence Scale (CEDVS) (83). The frequency, intensity and resolution subscales of the CPICS and items 2, 3, 4, 7, 8, and 9 of part one of the CEDVS were used to capture both emotional and physical forms of violence and conflict that children may witness between parents. Each has been shown to have good psychometric properties (84).

##### Teen dating violence

Adolescents reported on this using the Dating Abuse Perpetration Acts Scale (DAPAS) (85, 86). This measure examined the emotional, physical, and sexual aspects of teen dating violence (86).

##### Attitudes toward gender norms

All parents and teens completed all 24 items of the Gender-Equitable Men (GEM) Scale, developed in Brazil (87). It has been found to have reliability in a variety of LMICs (88, 89), including in Tanzania and Ghana (90).

#### Data analysis

For the quantitative data, R Statistical Computing was used (91). Cronbach's alpha coefficients were calculated for all scales and resultant coefficients ranged from 0.71 to 0.85, except for abusive parenting and child behaviour problems as reported by teens, where McDonald's Omega was calculated as this test better fit the data structure for these variables. This revealed the need to exclude certain items of the scale to increase the measures' reliability. Attitudes towards corporal or emotional punishment was reduced to a single item. Secondly, base 10 logarithmic transformations of physical IPV, positive parenting, abusive parenting, child behaviour problems reported by parents and co-parenting struggles, made the variables more normally distributed. One-way ANOVA and multi-level modelling were used to compare baseline and post-test results (70), except for emotional IPV. No transformations were able to make the emotional IPV data normally distributed, so a Wilcoxon signed rank test was performed on this variable. The significance criterion was set as 0.05, as is the usual convention (92). Cohen's *d* effect sizes were calculated for all variables except emotional IPV and were interpreted in terms of Cohen's criteria (93): effect sizes between 0.2 and 0.5 were categorised as small, those between 0.5 and 0.8 were categorised as medium, and those 0.8 or above as large.

For the qualitative data, thematic analysis was used as it provides a useful way to find patterns in data without a specific built-in theoretical underpinning (94). An interpretive approach was taken to this analysis which positioned the subjectivities of participants' everyday experiences at the heart of the analysis (95). The flexible step-by-step approach laid out by Braun and Clarke (95) was used, and qualitative data was manually coded. It was analysed by the first author under the supervision of the co-author. Translation was done similtaneously during the advisory board discussions. For the interviews, the research assistants transcribed and translated the recordings. The author familiarised herself with the transcripts in MS Word documents (95). Then, initial codes were generated. All transcripts were coded, where new codes were added and existing codes were refined. Once all was coded, the codes were collated into initial themes. Themes and codes were reviewed and refined, ensuring they were distinct and clear. Themes were named and an analytic narrative with extracts was created.

## Results

### Sample description

Of the 50 families recruited, 40 (80%) of the women were the biological mothers, and 42 (84%) were the biological fathers. One mother was a foster mother; otherwise, the sample included four step-parents, nine grandparents, one great-grandparent, and three cousins of the focal teens. Of the teens who participated, 31 (62%) were girls. As can be seen in Table 1, most (138, 92%) could read easily. Economic stress was evident in that 94 (94%) of the caregivers were unemployed, and only one (1%) was in full-time employment, although only one caregiver reported running out of money for food or other essentials in the past month. In terms of violence rates at baseline: 26% of women caregivers reported experiencing physical violence from a partner at baseline. All 50 women caregivers reported emotional violence from their partner. Perpetration was also reported as occurring commonly: 26% of women caregivers and 22% of male caregivers reported being physically violent at least once in the last four weeks. Of the teens, 46% reported their caregivers had been physically violent towards them at least once in the last four weeks. We retained all participants from pre to post-data collection.

**Table 1:** Sample description.

Socio-demographics	Adolescents (*N* = 50)	Women Caregivers (*N* = 50)	Male Caregiver (*N* = 50)	Total (*N* = 150)
Literacy				
Cannot read at all	4 (8%)	1 (2%)	1 (2%)	6 (4%)
Can read with a little difficulty	3 (6%)	2 (4%)	1 (2%)	6 (4%)
Can read easily	43 (86%)	47 (94%)	48 (96%)	138 (92%)
Employment Status				
Unemployed	–	48 (96%)	46 (92%)	94 (94%)
Self-employed	–	2 (4%)	2 (4%)	4 (4%)
Employed full-time		0 (0%)	1 (2%)	1 (1%)
Employed part-time	–	0 (0%)	1 (2%)	1 (1%)
Ran out of money for food or essentials in last month				
No	–	50 (100%)	49 (98%)	99 (99%)
Yes	–	0 (0%)	1 (2%)	1 (1%)
Caregivers’ relationship with teen				
Biological parent	–	40 (80%)	42 (84%)	82 (82%)
Stepparent	–	3 (6%)	1 (2%)	4 (4%)
Grandparent	–	5 (10%)	4 (8%)	9 (9%)
Great grandparent	–	0 (0%)	1 (2%)	1 (1%)
Cousin	–	1 (2%)	2 (4%)	3 (3%)
Foster parent	–	1 (2%)	0 (0%)	1 (1%)
Who lives in the household				
Both mother and father	42 (84%)			
Only mother	0 (0%)			
Only father	0 (0%)			
Neither mother nor father	8 (16%)			
Problems in the home				
An unwell adult	11 (22%)	13 (26%)	19 (38%)	43 (28.6%)
HIV/AIDS/COVID-19 death	4 (8%)	12 (24%)	14 (28%)	30 (20%)
People who drink or take drugs	18 (36%)	16 (32%)	18 (36%)	52 (34.6%)
An unwell child	7 (14%)	7 (14%)	9 (18%)	23 (15.3%)
A child with difficulties at school	26 (52%)	33 (66%	31 (62%)	90 (60%)

Participant attendance was extremely high. There was very little difference between the women, men and teens in terms of their attendance and overall average attendance was 96.59%. The men's attendance was slightly lower at 94.99%, while the women's and teens' attendance were almost the same. The average attendance across the sessions remained much the same, with no session attendance dropping below 92%. Session three had the highest attendance average and session one had the lowest attendance.

The results are summarised in Table 2. Notably, there was a reduction in the primary outcome, physical IPV (*F* = 9.61, *df* = 1, *p* = 0.003), with a medium effect size [*d* = −0.62; 95% CI (−1.03, −0.21)], even though the scores were relatively low at baseline. Emotional IPV scores, however, were high at baseline and did not decrease significantly. Women caregiver participants reported experiencing high levels of emotional IPV, often in the baseline survey (*m* = 22.06, SD = 3.39).

**Table 2:** Pre- and post-test statistics.

Outcome	Respondent	Mean (SD)	Median	Pre-test		Post-test		Effect Size^b^ [95% CI]
				Mean (SD)	Median	Test statistic(p)	*N*	
Overall physical intimate partner violence^c^/84^a^	**Women caregiver**	**1.84** (**3.98)**	**0**	**0.18** (**0.6)**	**0**	**9.612**(**<0.01)**	**50**	**−0.62 [−1.03, −0.21]**
Overall emotional intimate partner violence^d^/24	Women caregiver	22.06 (3.39)	24	21 (4.16)	24	461.5 (0.19)	50	
Parent intimate partner violence/56	Teen	5.40 (5.8)	4	4.14 (3.43)	2	1.76 (0.19)	50	−0.27 [−0.66, 0.13]
**Positive parenting** ^c^ **/80**	**All**	**71.3** (**8.14)**	**73**	**76.25** (**4.86)**	**77.5**	**35.82**(**<0.001)**	**150**	**0.69 [0.46, 0.92]**
**Abusive parenting** ^c^ ^,^ ^e^ **/72**	**All**	**3.75** (**5.1)**	**2**	**0.86** (**1.35)**	**0**	**68.25**(**<0.001)**	**150**	**−0.95 [−1.19, −0.71]**
Attitudes on corporal or emotional punishment/4	All	1.58 (1.22)	1	1.84 (1.62)	1	2.46 (0.12)	150	0.18 [−0.05, 0.41]
More equitable gender attitudes/27	**All**	**15.87** (**3.88)**	**15**	**18.13** (**4.18)**	**17**	**17.11** (**<0.001)**	**109**	**0.56 [0.29, 0.83]**
Parenting stress/56	**All**	**12.02** (**5.75)**	**14**	**5.64** (**3.59)**	**6**	**88.58** (**<0.001)**	**100**	**−1.33 [−1.62, −1.02]**
Co-parenting struggles^c^/78	**Both caregivers**	**5.21** (**7.85)**	**3**	**0.94** (**2.07)**	**0**	**56.01** (**<0.001)**	**100**	**−1.06 [−1.36, −0.76]**
Child Behaviour problems/28	**Both caregivers**	**2.59** (**3.06)**	**2**	**1.45** (**2.32)**	**0**	**8.08** (**<0.01)**	**100**	**−0.42 [0.70, −0.14]**
Child Behaviour problems^e^/50	**Teens**	**8.48** (**4.14)**	**7**	**6** (**2.93)**	**5**	**11.96** (**<0.001)**	**50**	**−0.69 [−1.10, −0.28]**

Statistically significant differences (*p* < 0.05) between pre- and post-tests are bolded.

a Value indicates the maximum total score.

b Cohen's d effect sizes based on comparisons between baseline and post-test scores. These are only recorded when the variable meets the assumptions of Cohen's *d*.

c indicates the variables that were transformed using logistic transformation before tests were performed.

d This indicates that a Wilcox signed rank test was used instead of an ANOVA test as the data for this variable was extremely skewed.

e This indicates that McDonald's Omega was calculated for reliability rather than Cronbach's alpha coefficient.

Of the variables theoretically linked to IPV, comparison between baseline and post-test scores showed a significant increase in equitable gender attitudes (*F* = 24.33, *df* = 1, *p* < 0.001). Equitable gender attitudes scores were relatively high at baseline (*m* = 15.87 and SD = 3.88), but even so, a medium effect size was found [*d* = 0.56% and 95% CI (0.39, 0.94)]. A comparison between baseline and post-test scores showed a significant reduction in co-parenting struggles (*F* = 56.01, *df* = 1, and *p* < 0.001), with a large effect size [*d* = −1.06% and 95% CI (−1.36, −0.76)]. Co-parenting struggle scores were relatively low at baseline ($\bar{x}$ = 5.21 and SD = 7.85). A comparison between baseline and post-test scores showed a non-significant reduction in parent IPV reported by teens (*F* = 1.76, *df* = 1, and *p* = 0.19). Parent IPV scores reported by teens were relatively low at baseline (*m* = 5.40 and SD = 5.77).

While reductions in IPV were the primary outcomes in this study, it was essential that the programme, centrally a parenting programme, also reduced VAC. A comparison between baseline and post-test scores showed a significant decrease in abusive parenting (*F* = 68.25, *df* = 1, and *p* < 0.001), with a large effect size [*d* = −0.95% and 95% CI (−1.19, −0.71)], even though abusive parenting scores were relatively low at baseline (*m* = 3.75 and SD = 5.10). Positive parenting was quite high at baseline (*m* = 71.32 and SD = 8.14), but nonetheless there was a significant increase in positive parenting (*F* = 35.82, *df* = 1, and *p* < 0.001), with a medium effect size [*d* = 0.69; 95% CI (0.46, 0.92)]. Comparison between baseline and post-test scores showed a non-significant increase in positive attitudes towards corporal or emotional punishment (*F* = 2.46, *df* = 1, and *p* = 0.12), and a negligible effect size [*d* = −0.18% and 95% CI (−0.05, 0.41)]. Positive attitudes towards corporal or emotional punishment were extremely low at baseline (*m* = 1.58 and SD = 1.22), but surprisingly increased over time. There was a significant decrease in parenting stress between baseline and post-test (*F* = 88.58, *df* = 1, and *p* < 0.001), with a large effect size [*d* = −1.33% and 95% CI (−1.64, −1.02)]. Parenting stress had been high at baseline (*m* = 12.02 and SD = 5.75), and is the variable on which most change was observed.

Parents reported a significant decrease in child behaviour problems (*F* = 8.08, *df* = 1, and *p* < 0.001), albeit with a small effect size [*d* = −0.42% and 95% CI (−0.70, −0.14)]. Teens similarly reported a decrease in behaviour problems (*F* = 11.96, *df* = 1, and *p* < 0.001), with a medium effect size [*d* = −0.69% and 95% CI (−1.10, −0.28)]. The baseline child behaviour problems reported by teens were much higher than the parent report (*m* = 8.48 and SD = 4.14).

Sensitivity analyses were carried out to explore whether participant status (male caregiver, woman caregiver or teen) or being in a particular programme group affected the outcomes. Participant status did not affect outcomes, but programme group had effects on changes in emotional IPV, gender attitudes, parenting stress, and teen-reported child behaviour problems over time. For emotional intimate partner violence reported by women, this analysis found that which programme group the participant was in had a role to play in how much emotional intimate partner violence decreased or increased between baseline and post-test. The programme group variable may reflect area, group dynamics, and facilitation. It is unfortunately not possible to disentangle these, and future research should explore these dynamics further.

### Qualitative findings

Participant interviews and the facilitator focus group described participants' experiences of the benefits of participation, barriers to participation, and enablers and barriers to engaging men in the programme.

#### Benefits

Firstly, all participants, including the facilitators, emphasised that the programme improved the relationship between both parents and their children.

“Yes, my relationship with my parents is better now. We share stories or jokes with my parents, we do physical exercise together with my parents, we do a family budget, we share responsibilities at home” (Teen, 14 years old).

“They (parent participants) were waiting for the school to do that or discipline their children, or they were able to do that, telling other family members to unite them. But because of this program, they are now able to talk as a family, planning their own things as a family” (facilitator).

They also reflected that it had a positive effect on co-parenting partners' relationships.

“Yes, my relationship with my partner has been greatly improved because we do family budget together, we do physical exercise together, we listen to each other, we eat together, we share jokes and stories together, hence there are no more conflicts or fighting or tension” (woman caregiver, 47 years old, biological mother).

Participants reported that the programme improved relationships beyond the co-parenting partners' relationship and parent-child relationships: teens spoke about improved relationships with their friends and other teens, and many participants spoke about improved relationships with relatives in their wider families, and in their community.

“The programme was good since we were taught about aspects which improve the way we live as a family and the way we associate with the community” (male caregiver, 43 years old, biological father).

Participants highlighted that after the programme, their families did things together as a family which they had not been doing before the programme. One woman caregiver said,

“Yes, this programme has improved my relationship with my partner and my child. Both of us now know what we are expected to do at home like duties and responsibilities hence cases of fighting or misunderstanding are less. We do family budget together with my family and we agree together about the final results or decisions that we made on family budget peacefully” (woman caregiver, 39 years old, biological mother).

The most common activity that was new to the families was related to budget and planning, covered in session seven of the programme. They particularly highlighted that they had started to budget together as a family, and tried to take all family members' needs and wishes into consideration.

“Before the programme, we didn’t plan things together and I could even buy needs and wants without the knowledge of other family members. The programme helped to build a stronger relationship between our family and as for now we now know that before purchasing anything we must engage in a family budget whereby every family member is required to give suggestions and ideas” (male caregiver, 34 years old, biological father).

It seemed that families were using budgeting skills in tandem with the other elements of the programme (such as one-on-one time, praising, sharing responsibilities as a family) to work together as a family and include everyone's ideas. Many highlighted that Session 5, which dealt with family routines, had helped to reduce conflict because everyone knew what their responsibilities were. The facilitators echoed these sentiments and suggested that programme's emphasis on doing things together and planning together had helped challenge traditional norms around the lower status of children within families.

A few participants spoke directly to a change in their attitudes toward gender norms.

“We do some jobs which are not meant for us as fathers” (male caregiver, 58 years old, biological father)

“I learnt that no duty is meant for boys or girls only but both of us, can do all the duties” (Teen, 13 years old).

Others were more subtle. They directly indicated that they felt that they could now do things that were seen as for the other gender. When reflecting on more tangible benefits of the programme, they hinted at more subtle changes in their gendered ideas surrounding parenting. For instance, one male caregiver suggested that the programme had made them more available for their daughters and disrupted a gender norm around the role of fathers as only breadwinners.

### Enablers

The participants were overwhelmingly positive about the programme.

“We really appreciate this programme, and the programme treated us in a positive way and we’re so grateful and delighted about this programme” (woman caregiver, 54 years old, biological mother).

Participants reported liking all the sessions equally and that nothing should be left out if the programme were to be implemented again. Facilitators said participants particularly enjoyed the content on one-on-one time, budgeting and saving, sharing responsibilities, the mapping of safe and unsafe places in the community, solving problems in the family and managing anger and stress. They also highlighted that group discussions and role plays were the most enjoyed activities.

One male caregiver appreciated that the programme included both women and men equally and actively. He said,

“The most essential aspects in this programme include that of preserving our cultural norms and values, respecting each other and that of inclusiveness of every gender within families and societies” (male caregiver, 45 years old, biological father).

Some participants highlighted that they were keen to share what they had learnt from the programme with other people, which motivated them to attend. Two participants also highlighted, as in the above quote from the male caregiver, that they learned about the culture and traditions in the community where they live, which helped to reduce conflicts at home or in their community and encouraged them to keep attending.

### Barriers

Some participants highlighted a few barriers to their participation, but the majority said that there were no barriers to their participation. Events that had interrupted participation included a death in the family, which meant that they had to miss one or two sessions. Some participants reported a long travel time to get to where the sessions were. Facilitators concurred with these challenges and added that church events had sometimes made it difficult for participants to attend.

Facilitators also highlighted that at first, teens felt scared to share their ideas with their parents in the room. Yet they did suggest that as the weeks progressed, teens became more confident and engaged more, contrary to cultural norms. One facilitator said,

“Due to our cultural beliefs, when parents or elderly people are having a discussion, usually children are sent out. So, it was something that they were afraid to share their minds whilst their parents were around” (facilitator).

The session focusing on how to talk about sex with teens was reported to be difficult in some groups, but it transpired that these groups had not separated men, women and teens, as suggested in the manual for this session, and some participants felt that discussing sex in front of their children would encourage their children to get a sexual partner. In the groups that followed the manual, facilitators reported that the participants felt free to speak their minds, and that learning and acceptance happened. However, when they brought the larger group together for feedback after the separate discussions, they did have to be careful with the questions that they asked.

### Engaging men in the programme

#### Barriers

Facilitators spoke of initial resistance from male caregivers that made it difficult to conduct the first three sessions, especially on the topic of making rules together as a family. They said male caregivers felt the program was coming to take away their authority as the head of the household who traditionally set the rules. However, facilitators reported that as the male caregivers participated, they started to appreciate the benefits of doing things together as a family. One facilitator said,

“Sessions one, two and three were initially difficult because the fathers actually said that this program is coming to destroy our families. But as they went on and remained part of the program, they then said ‘Ohh! Wow! but this program is actually coming to build the family’” (facilitator).

#### Enablers

The facilitators highlighted that despite some initial resistance from the male caregivers, they remained active participants in all the sessions. The role plays and the “Sinovuyo Buddies” were important in keeping men engaged. One male caregiver said,

“I liked all the sessions but the aspect of emotions proved to be a challenge to me but I kept on revisiting it and I gained assistance from my Sinovuyo buddy after consulting him” (male caregiver, 43 years, biological father).

Facilitators also emphasised that the male caregivers particularly enjoyed the topic of sharing responsibilities. One facilitator said,

“There is a role play in the manual, and they (the male caregivers) really like the role play. They are saying that because of that role-play, the fathers were able to see there was a need to help out mothers with the house chores. Uh, even in caring for the children. In the role play, it stated that a woman who is being helped out by his spouse is healthier than one who is overloaded by work. So, they loved that part since they didn’t want the community to start saying, so if my wife is looking weak, that means I'm not able to take care of her. That’s why they loved the sharing of responsibilities topic” (facilitator).

## Discussion

Overall, the findings of this study suggest that this programme is promising for the prevention of both VAW and VAC. This programme led to encouraging positive outcomes which were demonstrated both qualitatively and quantitatively. It led to significant reductions in physical IPV, co-parenting struggles, abusive parenting, parenting stress, and teen behaviour problems reported on by both teens and parents separately and significant increases in positive parenting and equitable gender attitudes. These quantitative findings are supported by the qualitative findings. Participants and facilitators highlighted several key benefits, including improved relationships between parents and teens and between co-parenting partners and between themselves and their community, families now doing things together, less conflict and tension in the home, and changes to gender attitudes. Discussions of gender equity material were initially not acceptable, but, as with positive parenting, once participants saw the benefits, change began to happen.

These findings contribute to the currently limited body of evidence that suggests that a parenting programme can help prevent VAW and VAC (43–45). For example, a recent programme integrating parenting and nutrition interventions was found to yield the greatest improvements in maternal and paternal parenting practices as well as early childhood development outcomes (96, 97). This study's findings also suggest that intervening in co-parenting and in gender attitudes in families may lead to significant reductions in the experiencing and perpetration of both abusive parenting and IPV. While a recent systematic review found that co-parenting programmes have a small but significant positive effect on parents' well-being, the child's adjustment and the parent-child relationship and the relationship between parents (37), this study found that a parenting programme with an overarching focus on co-parenting can have a significant impact on violence perpetration.

The findings of this study suggest several key aspects to consider in the work to prevent both VAW and VAC.

Firstly, trained and motivated local facilitators are crucial. This programme was implemented by local community members who were well-trained by Clowns Without Borders South Africa and supported by ongoing coaching. We posit that a large part of the reported positivity for the programme from the participants comes from the fact that the facilitators were both well-known to the participants and had the training to create safe, comfortable spaces for the participants in the sessions. The quantitative results that reductions in emotional IPV depended on group suggest that the facilitator may have a key role in some changes that the programme made. Findings of other studies suggest that facilitators' gender attitudes play a key role in the shifts made by a programme (56, 98). Boyer and colleagues (2022) (99) found that improvements associated with their IPV prevention programme were greater with facilitators with more gender equitable attitudes at baseline. The training for the programme reported in this paper did not include an intensive focus on gender equality and power theory, but rather focused on helping facilitators to be able to create a safe, comfortable environment for participants to share and explore the content which included gender attitudes. We feel this environment allowed facilitators to avoid resistance from participants.

Secondly, we suggest that the programme's focus on small, achievable shifts in gender attitudes through the choice of a light-touch approach to addressing gender transformation may have helped reduce the risk of resistance. The adaptation of this programme focused on sparking discussions in families and the larger community about more equal gender attitudes, rather than insisting on change. A concern before implementation was that participants, especially male caregivers, would outright reject the gender transformative components and drop out of the programme. Happily, the programme was very well received by the male caregivers. Even when there was some initial resistance to the programme, men continued to engage and participate and soon were able to see the benefits of participating.

At the same time, there was a reduction in physical IPV but not emotional IPV, suggesting the need to explore and strengthen programmes' ability to address the complexity of emotional IPV. The programme was originally designed as a parenting programme, and the parenting-related outcomes saw the greatest effect. This suggests that VAW may be a more difficult behaviour to change, or that more content, emphasis and time should be given to the couple's relationship. More work is needed to understand IPV prevention, especially emotional IPV prevention, within this context.

This programme created many different spaces for participants to share their feelings and ideas. Giving men, women, and children a space to discuss amongst themselves as well as having larger group and family unit discussions is critical. The use of three facilitators with at least one male facilitator was crucial to this. The three facilitators could each take one of the three participant groups, one men's group, one women's group and one teens' group. This was found to be a successful strategy in the Ugandan Parenting for Respectability programme which managed to recruit and retain a majority of their male participants (100). Additionally, the programme also had sections where the participants were divided into their family units for certain activities, especially practising novel skills such as praising one another. This was already established as part of the original programme, but the inclusion of the male caregiver in these family-only activities may increase the likelihood that the skills would be used in future, since both adults were aware of the skills. The participants also had the chance to share with the whole group of participants. This allowed for teens and both male and woman caregivers to learn from one another, and families to learn from each other. Our findings here also highlight the importance of implementation fidelity to avoid disengagement, especially by male caregivers.

Lastly, feedback from participants and stakeholders shows that there is a need to layer this programme within existing services, specifically within income generation projects. This has been highlighted both as helping to potentially increase the benefits and power of the impact on families and as an important tool to help increase the engagement of men. A recent example of this for preventing VAC is a parallel cluster randomised controlled trial which examined the combined and separate effects of parenting and economic strengthening programmes in farming communities in Tanzania. They found that combining parenting and economic strengthening programmes was most effective compared to either alone (101).

### Limitations

As with any other study, there were limitations. Firstly, causality could not be established because of the pre-post uncontrolled design of this study. Secondly, the results may not be generalisable to other populations, given its focus and specific location. Thirdly, there was a very short follow-up period for data collection, which did not allow for the assessment of its long-term effect. Fourthly, the self-reported nature of the study's measures may mean that some social desirability bias was introduced. This study also had a small sample size which means that mediators and moderators of the effects of this programme could not be investigated. These are unavoidable limitations of a pre-post pilot design (64). Additionally, the qualitative component had a small sample, which may have resulted in a lack of diverse experiences. Lastly, there were relatively low levels of VAC and VAW in this sample so we do not know how this programme could perform in the context of higher levels. These low levels may be genuine, or may be due to social desirability bias, given that research assistants were from the local community and would have been familiar with the participants.

### Future research

Findings from this small pre-post pilot of the programme were positive, suggesting that there would be value in studying this programme with a larger sample using a more robust evaluation such as a randomised controlled trial. Given the context of low rates of VAW and VAC, future studies should test the programme in contexts of higher rates of violence. In addition, the findings highlight the need to explore further the role of facilitators especially regarding shifting negative gender attitudes.

## Conclusions

Results suggest that there is merit in pursuing the goal of preventing both VAW and VAC in the same parenting programme. Along with significant reductions in both forms of violence, this study suggests that this programme leads to reductions in co-parenting struggles, abusive parenting, parenting stress, and teen behaviour problems, and increases in positive parenting and equitable gender attitudes. Participants and facilitators spoke to benefits, including improved relationships between parents and teens and between co-parenting partners and between themselves and their community, families now doing things together, less conflict and tension in the home, and changes to gender attitudes. By working with teenagers and their parents, in an integrated approach to preventing family violence, this action-orientated and policy-relevant study shows that it is possible for a parenting programme to prevent both VAW and VAC simultaneously (102).

## Data Availability

The datasets presented in this study can be found in online repositories. The names of the repository/repositories and accession number(s) can be found below: The dataset(s) and relevant materials supporting the conclusions of this article is(are) available in the Zivahub repository, https://doi.org/10.25375/uct.28661627

## References

[1] Garcia-Moreno C Pallitto C Devries K Stockl H Watts C Abrahams N. Global and Regional Estimates of Violence Against Women: Prevalence and Health Effects of Intimate Partner Violence and Non-Partner Sexual Violence. United States of America: World Health Organisation (2013).

[2] Hillis S Mercy J Amobi A Kress H. Global prevalence of past-year violence against children: a systematic review and Minimum estimates. Pediatrics. (2016) 137(3):1–13. 10.1542/peds.2015-4079 PMC6496958 26810785

[3] Namy S Carlson C O'Hara K Nakuti J Bukuluki P Lwanyaaga J. Towards a feminist understanding of intersecting violence against women and children in the family. Soc Sci Med. (2017) 184:40–8. 10.1016/j.socscimed.2017.04.042 28501019 PMC5737762

[4] Carlson C Namy S Norcini Pala A Wainberg ML Michau L Nakuti J. Violence against children and intimate partner violence against women: overlap and common contributing factors among caregiver-adolescent dyads. BMC Public Health. (2020) 20(1):124. 10.1186/s12889-019-8115-0 31996179 PMC6988249

[5] Pearson I Stockl H Guedes A Meinck F Page S Gennari F. The co-occurrence of intimate partner violence and violence against children: a systematic review on associated factors in low- and middle-income countries. Trauma Violence Abuse. (2023) 24(4):2097–114. 10.1177/15248380221082943 35481390 PMC10486154

[6] Falb KL Blackwell A Hategekimana JD Sifat M Roth D O'Connor M. Co-occurring intimate partner violence and child abuse in eastern democratic Republic of Congo: the influence of early life experiences of abuse. Violence Against Women (2022) 30(3-4):873–89. 10.1177/10778012221145302 36579814

[7] Zimbabwe National Statistics Agency I, UNICEF I. Zimbabwe multiple indicator cluster survey 2019: Survey Findings Report (2019).

[8] Chigiji H Fry D Mwadiwa TE Elizalde A Izumi N Baago-Rasmussen L. Risk factors and health consequences of physical and emotional violence against children in Zimbabwe: a nationally representative survey. BMJ Glob Health. (2018) 3(3):e000533. 10.1136/bmjgh-2017-000533 29989051 PMC6035512

[9] Lessard G Alvarez-Lizotte P. The exposure of children to intimate partner violence: potential bridges between two fields in research and psychosocial intervention. Research and interventions often focus on a specific form of violence without considering other forms of victimization. Child Abuse Negl. (2015) 48:29–38. 10.1016/j.chiabu.2015.05.004 26003820

[10] Slep AM O'Leary SG. Parent and partner violence in families with young children: rates, patterns, and connections. J Consult Clin Psychol. (2005) 73(3):435–44. 10.1037/0022-006X.73.3.435 15982141

[11] Hamby S Finkelhor D Turner H Ormrod R. The overlap of witnessing partner violence with child maltreatment and other victimizations in a nationally representative survey of youth. Child Abuse Negl. (2010) 34(10):734–41. 10.1016/j.chiabu.2010.03.001 20850182

[12] Dekel B Abrahams N Andipatin M. Exploring the intersection between violence against women and children from the perspective of parents convicted of child homicide. J Fam Violence. (2019) 34(1):9–20. 10.1007/s10896-018-9964-5 30686856 PMC6325095

[13] Norman R Bradshaw D Schneider M Jewkes R Matthews S Abrahams N. Estimating the burden of disease attributable to interpersonal violence in South Africa in 2000. S Afr Med J. (2007) 97(8):653–6. Available online at: https://pubmed.ncbi.nlm.nih.gov/17957838/17957838

[14] Machisa MT Christofides N Jewkes R. Structural pathways between child abuse, poor mental health outcomes and male-perpetrated intimate partner violence (ipv). PLoS One. (2016) 11(3):1–15. 10.1371/journal.pone.0150986 PMC4795913 26986056

[15] Ward CL Artz L Leoschut L Kassanjee R Burton P. Sexual violence against children in South Africa: a nationally representative cross-sectional study of prevalence and correlates. Lancet Glob Health. (2018) 6(4):e460–e8. 10.1016/s2214-109x(18)30060-3 29530424

[16] Hughes K Bellis MA Hardcastle KA Sethi D Butchart A Milton C. The effect of multiple adverse childhood experiences on health: a systematic review and meta-analysis. Lancet Public Health. (2017) 2(8):e356–e66. 10.1016/S2468-2667(17)30118-4 29253477

[17] McTavish JR MacGregor JC Wathen CN MacMillan HL. Children’s exposure to intimate partner violence: an overview. Int Rev Psychiatry. (2016) 28(5):504–18. 10.1080/09540261.2016.1205001 27414209

[18] MacMillan HL Wathen CN. Children’s exposure to intimate partner violence. Child Adolesc Psychiatr Clin N Am. (2014) 23(2):295–308. 10.1016/j.chc.2013.12.008 24656581

[19] Holt S Buckley H Whelan S. The impact of exposure to domestic violence on children and young people: a review of the literature. Child Abuse Negl. (2008) 32(8):797–810. 10.1016/j.chiabu.2008.02.004 18752848

[20] Hazen AL Connelly CD Kelleher KJ Barth RP Landsverk JA. Female caregivers’ experiences with intimate partner violence and behavior problems in children investigated as victims of maltreatment. Pediatrics. (2006) 117(1):99–109. 10.1542/peds.2004-2542 16396866

[21] Fieggen AG Wiemann M Brown C Van As AB Swingler GH Peter JC. Inhuman shields: children caught in the crossfire of domestic violence. S Afr Med J. (2004) 94(4):293–6. Available online at: https://pubmed.ncbi.nlm.nih.gov/15150945/15150945

[22] Gondolf EW. The victims of court-ordered batterers: their victimization, helpseeking, and perceptions. Violence Against Women. (2016) 5(6):659–76.

[23] Chiesa AE Kallechey L Harlaar N Rashaan Ford C Garrido EF Betts WR. Intimate partner violence victimization and parenting: a systematic review. Child Abuse Negl. (2018) 80:285–300. 10.1016/j.chiabu.2018.03.028 29665506

[24] Nicklas E Mackenzie MJ. Intimate partner violence and risk for child neglect during early childhood in a community sample of Fragile families. J Fam Violence. (2012) 28(1):17–29. 10.1007/s10896-012-9491-8

[25] Postmus JL Huang C-C Mathisen-Stylianou A. The impact of physical and economic abuse on maternal mental health and parenting. Child Youth Serv Rev. (2012) 34(9):1922–8. 10.1016/j.childyouth.2012.06.005

[26] Guedes A Bott S Garcia-Moreno C Colombini M. Bridging the gaps: a global review of intersections of violence against women and violence against children. Glob Health Action. (2016) 9(1):1–15. 10.3402/gha.v9.31516 PMC4916258 27329936

[27] Sijtsema JJ Stolz EA Bogaerts S. Unique risk factors of the co-occurrence between child maltreatment and intimate partner violence perpetration. Eur Psychol. (2020) 25(2):122–33. 10.1027/1016-9040/a000396

[28] Bott S Ruiz-Celis AP Mendoza JA Guedes A. Correlates of co-occurring physical child punishment and physical intimate partner violence in Colombia, Mexico and Peru. BMC Public Health. (2022) 22(1):2195. 10.1186/s12889-022-14453-6 36443733 PMC9702951

[29] Bruhl A Ward CL Lachman JM Foran HM Raleva M Baban A. Co-Occurrence of intimate partner violence against mothers and maltreatment of their children with behavioral problems in Eastern Europe. Violence Against Women. (2023) 29(12-13):2439–63. 10.1177/10778012231188090 37475529 PMC10496420

[30] Gracia E Rodriguez CM Martin-Fernandez M Lila M. Acceptability of family violence: underlying ties between intimate partner violence and child abuse. J Interpers Violence. (2017) 35(17-18): 3217–36. 10.1177/0886260517707310 29294751

[31] Bidarra ZS Lessard G Dumont A. Co-Occurrence of intimate partner violence and child sexual abuse: prevalence, risk factors and related issues. Child Abuse Negl. (2016) 55:10–21. 10.1016/j.chiabu.2016.03.007 27060785

[32] Wilkins N Tsao B Hertz M Davis R Klevens J. Connecting the Dots: An Overview of the Links among Multiple Forms of Violence is a Publication of the Centers for Disease Control and Prevention and Prevention Institute. Oakland, CA: Prevention Institute (2014).

[33] Pearson S Makadzange P. Help-Seeking behaviour for sexual-health concerns: a qualitative study of men in Zimbabwe. Cult Health Sex. (2008) 10(4):361–76. 10.1080/13691050801894819 18484379

[34] Mugweni E Pearson S Omar M. Traditional gender roles, forced sex and hiv in Zimbabwean marriages. Cult Health Sex. (2012) 14(5):577–90. 10.1080/13691058.2012.671962 22472019

[35] Chitsike C. Culture as a barrier to rural women’s entrepreneurship: experience from Zimbabwe. Gend Dev. (2000) 8(1):71–7. 10.1080/741923408 12349641

[36] Katz LF Low SM. Marital violence, co-parenting, and family-level processes in relation to children’s adjustment. J Fam Psychol. (2004) 18(2):372–82. 10.1037/0893-3200.18.2.372 15222844

[37] Eira Nunes C De Roten Y El Ghaziri N Favez N Darwiche J. Co-parenting programs: a systematic review and meta-analysis. Fam Relat. (2025) 70(3):759–76. 10.1111/fare.12438

[38] Feinberg ME Kan ML. Establishing family foundations: intervention effects on coparenting, parent/infant well-being, and parent-child relations. J Fam Psychol. (2008) 22(2):253–63. 10.1037/0893-3200.22.2.253 18410212 PMC3178882

[39] Feinberg ME Jones DE Hostetler ML Roettger ME Paul IM Ehrenthal DB. Couple-Focused prevention at the transition to parenthood, a randomized trial: effects on coparenting, parenting, family violence, and parent and child adjustment. Prev Sci. (2016) 17(6):751–64. 10.1007/s11121-016-0674-z 27334116 PMC10351206

[40] Ghoshal R Douard A-C Sikder S Roy N Saulnier D. Risk and protective factors for ipv in low- and middle-income countries: a systematic review. J Aggress Maltreat Trauma. (2023) 32(4):505–22. 10.1080/10926771.2022.2154185

[41] Vlahovicova K Melendez-Torres GJ Leijten P Knerr W Gardner F. Parenting programs for the prevention of child physical abuse recurrence: a systematic review and meta-analysis. Clin Child Fam Psychol Rev. (2017) 20(3):351–65. 10.1007/s10567-017-0232-7 28378136 PMC5527061

[42] McCoy A Melendez-Torres GJ Gardner F. Parenting interventions to prevent violence against children in low- and middle-income countries in east and Southeast Asia: a systematic review and multi-level meta-analysis. Child Abuse Negl. (2020) 103:104444. 10.1016/j.chiabu.2020.104444 32171126

[43] Barlow J. Preventing child maltreatment and youth violence using parent training and home-visiting programmes. In: Donnelly PD Ward CL, editors. Oxford Textbook of Violence Prevention. Oxford: Oxford University Press (2015). p. 133–140.

[44] Lundgren R Amin A. Addressing intimate partner violence and sexual violence among adolescents: emerging evidence of effectiveness. J Adolesc Health. (2015) 56(1):S42–50. 10.1016/j.jadohealth.2014.08.012 25528978

[45] Bacchus LJ Colombini M Urbina MC Howarth E Gardner F Annan J. Exploring opportunities for coordinated responses to intimate partner violence and child maltreatment in low and middle income countries: a scoping review. Psychol Health Med. (2017) 22(1):135–65. 10.1080/13548506.2016.1274410 28150500

[46] Bacchus LJ Colombini L Pearson I Gevers A Stöckl H Guedes A. Interventions that prevent or respond to intimate partner violence against women and violence against children: a systematic review. Lancet. (2024) 9(5):e326–e38. 10.1016/S2468-2667(24)00048-3 38702097

[47] World Health Organization I. Respect - Seven Strategies to Prevent Violence Against Women. Geneva: World Health Organization (2019).

[48] World Health Organization I. Inspire: Seven Strategies for Ending Violence Against Children. Geneva: World Health Organization (2018).

[49] Smith TK Duggan A Bair-Merritt MH Cox G. Systematic review of Fathers’ involvement in programmes for the primary prevention of child maltreatment. Child Abuse Rev. (2012) 21(4):237–54. 10.1002/car.2195

[50] Sarkadi A Kristiansson R Oberklaid F Bremberg S. Fathers’ involvement and children’s developmental outcomes: a systematic review of longitudinal studies. Acta Paediatr. (2008) 97(2):153–8. 10.1111/j.1651-2227.2007.00572.x 18052995

[51] Doyle K Swan M Manji S Daelmans B Greene M Chaudhury S. Nurturing Care and Men’s Engagement: Thematic Brief. New York: UNICEF (2022).

[52] Gonzalez JC Klein CC Barnett ML Schatz NK Garoosi T Chacko A. Intervention and implementation characteristics to enhance father engagement: a systematic review of parenting interventions. Clin Child Fam Psychol Rev. (2023) 26(2):445–58. 10.1007/s10567-023-00430-x 36947287 PMC10031187

[53] Panter-Brick C Burgess A Eggerman M McAllister F Pruett K Leckman JF. Practitioner review: engaging fathers–recommendations for a game change in parenting interventions based on a systematic review of the global evidence. J Child Psychol Psychiatry. (2014) 55(11):1187–212. 10.1111/jcpp.12280 24980187 PMC4277854

[54] Hawkins AJ Dollahite DC. Generative Fathering. Thousand Oaks: SAGE publications (1997).

[55] Lechowicz ME Jiang Y Tully LA Burn MT Collins DAJ Hawes DJ. Enhancing father engagement in parenting programs: translating research into practice recommendations. Aust Psychol. (2018) 54(2):83–9. 10.1111/ap.12361

[56] Kohli A Doyle K Uysal J Ojamuge D Karamage E Lundgren R. Building a theory of change to guide fatherhood programming to prevent family violence: a comparison of two programs. BMC Public Health. (2025) 25(1):3576. 10.1186/s12889-025-24883-7 41126098 PMC12542366

[57] Cluver LD Meinck F Steinert JI Shenderovich Y Doubt J Herrero Romero R. Parenting for lifelong health: a pragmatic cluster randomised controlled trial of a non-commercialised parenting programme for adolescents and their families in South Africa. BMJ Glob Health. (2018) 3(1):e000539. 10.1136/bmjgh-2017-000539 29564157 PMC5859808

[58] Janowski RK Wessels I Bojo S Monday F Maloney K Achut V. Transferability of evidence-based parenting programs to routine implementation in postconflict South Sudan. Res Soc Work Pract. (2020) 30(8):858–69. 10.1177/1049731520932986

[59] Liu S Xia X Lachman JM Zhang H. A feasibility study of parenting for lifelong health for adolescents in China. Res Soc Work Pract. (2024) 34(6):687–700. 10.1177/10497315241238964

[60] Jocson RM Alampay LP Lachman JM Maramba DHA Melgar ME Ward CL. Pre-Post mixed methods study of a parent and teen support intervention to prevent violence against adolescents in the Philippines. J Adolesc Health. (2023) 73(1):102–9. 10.1016/j.jadohealth.2023.02.027 37086250

[61] Muyambo B Ranga D. Socio-Economic impacts of labour migration from Zimbabwe to South Africa: an investigation based on rural bikita district. Migr Dev. (2020) 9(2):274–90. 10.1080/21632324.2019.1603670

[62] 2022 Population and Housing Census Data [Internet]. Open data for Africa (2022). Available online at: https://zimbabwe.opendataforafrica.org/arfkbif/zimbabwe-population-census-2022?regionId=ZW-MA (Accessed June 22, 2024).

[63] Doyle L Brady AM Byrne G. An overview of mixed methods research – revisited. J. Res. Nurs. (2016) 21:623–35. 10.1177/1744987116674257

[64] Craig P Dieppe P Macintyre S Michie S Nazareth I Petticrew M. Developing and evaluating complex interventions: the new medical research council guidance. Br Med J. (2008) 337(1655):1–6. 10.1136/bmj.a1655 PMC2769032 18824488

[65] Tortolero SR Markham CM Parcel GS Jr. Peters RJ Escobar-Chaves SL Basen-Engquist K. Using intervention mapping to adapt an effective hiv, sexually transmitted disease, and pregnancy prevention program for high-risk minority youth. Health Promot Pract. (2005) 6(3):286–98. 10.1177/1524839904266472 16020623

[66] Davidson N Booij A Ward CL. Could a parenting program be adapted to address both violence against children and against women? Views from stakeholders in Zimbabwe. J Fam Violence. (2026) 1(1):1–13. 10.1007/s10896-026-01082-5

[67] Sim A Annan J Puffer E Salhi C Betancourt T. Building Happy Families: Impact Evaluation of a Parenting and Family Skills Intervention for Migrant and Displaced Burmese Families in Thailand. New York, America: International Rescue Committee (2014).

[68] Puffer ES Green EP Chase RM Sim AL Zayzay J Friis E. Parents make the difference: a randomized-controlled trial of a parenting intervention in Liberia. Global Mental Health. (2015) 2(e15):1–15. 10.1017/gmh.2015.12 PMC5269617 28596863

[69] Sanders MR Kirby JN Tellegen CL Day JJ. The triple P-positive parenting program: a systematic review and meta-analysis of a multi-level system of parenting support. Clin Psychol Rev. (2014) 34(4):337–57. 10.1016/j.cpr.2014.04.003 24842549

[70] Terre Blanche M Durrheim K Painter D. Research in Practice: Applied Methods for the Social Services. Cape Town, South Africa: Juta and Company (2014).

[71] Guthrie G. Basic Research Methods: An Entry to Social Science Research. New Dehli, India: SAGE Publications (2010). p. 118–28.

[72] Straus MA Douglas EM. A short form of the revised conflict tactics scales, and typologies for severity and mutuality. Violence Vict. (2004) 19(5):507–20. 10.1891/088667004780927800 15844722

[73] Ellsberg M Heise L. Researching Violence Against Women: A Practical Guide for Researchers and Activists. Geneva: World Health Organization, PATH (2005).

[74] Decker MR Latimore AD Yasutake S Haviland M Ahmed S Blum RW. Gender-Based violence against adolescent and young adult women in low- and middle-income countries. J Adolesc Health. (2015) 56(2):188–96. 10.1016/j.jadohealth.2014.09.003 25620301

[75] Cluver LD Meinck F Yakubovich A Doubt J Redfern A Ward C. Reducing child abuse amongst adolescents in low- and middle-income countries: a Pre-post trial in South Africa. BMC Public Health. (2016) 16(1):567. 10.1186/s12889-016-3262-z 27919242 PMC5137206

[76] Meinck F Boyes ME Cluver L Ward CL Schmidt P DeStone S. Adaptation and psychometric properties of the ispcan child abuse screening tool for use in trials (icast-trial) among South African adolescents and their primary caregivers. Child Abuse Negl. (2018) 82:45–58. 10.1016/j.chiabu.2018.05.022 29860107

[77] Essau CA Sasagawa S Frick PJ. Psychometric properties of the Alabama parenting questionnaire. J Child Fam Stud. (2006) 15(5):595–614. 10.1007/s10826-006-9036-y

[78] Berry JO Jones WH. The parental stress scale: initial psychometric evidence. J Soc Pers Relat. (1995) 12(3):463–72. 10.1177/0265407595123009

[79] Feinberg ME Brown LD Kan ML. A multi-domain self-report measure of coparenting. Parent Sci Pract. (2012) 12(1):1–21. 10.1080/15295192.2012.638870 23166477 PMC3499623

[80] Carvalho TR Barham EJ Souza CD Böing E Crepaldi MA Vieira ML. Cross-Cultural adaptation of an instrument to assess coparenting: coparenting relationship scale. Psico-USF Bragança Paulista. (2018) 23(2):215–27. 10.1590/1413-82712018230203

[81] Rush AJ First MB Blacker D. Handbook of Psychiatric Measures. Arlington, VA: American Psychiatric Publishing (2009).

[82] Grych JH Seid M Fincham FD. Assessing marital conflict from the child’s perspective: the children’s perception of interparental conflict scale. Child Dev. (1992) 63(3):558–72. 10.2307/1131346 1600822

[83] Edleson JL Shin N Johnson-Armendariz KK. Measuring children’s exposure to domestic violence: the development and testing of the child exposure to domestic violence (cedv) scale. Child Youth Serv Rev. (2008) 30(5):502–21. 10.1016/j.childyouth.2007.11.006

[84] Ravi KE Tonui BC. A systematic review of the child exposure to domestic violence scale. Br J Soc Work. (2019) 50(1):101–18. 10.1093/bjsw/bcz028

[85] Goncy EA Rothman EF. The reliability and validity of the dating abuse perpetration acts scale in an urban, emergency department-based sample of male and female youth. J Interpers Violence. (2019) 34(11):2246–68. 10.1177/0886260516660299 27443413

[86] Rothman EF Stuart GL Winter M Wang N Bowen DJ Bernstein J. Youth alcohol use and dating abuse victimization and perpetration: a test of the relationships at the daily level in a sample of pediatric emergency department patients who use alcohol. J Interpers Violence. (2012) 27(15):2959–79. 10.1177/0886260512441076 22550149 PMC3678358

[87] Pulerwitz J Barker G. Measuring attitudes toward gender norms among young men in Brazil: development and psychometric evaluation of the Gem scale. Men Masc. (2008) 10(3):322–38. 10.1177/1097184X06298778

[88] Sayem AM Nury ATMS. An assessment of attitude towards equitable gender norms among muslim women in Bangladesh. Womens Stud Int Forum. (2013) 40:102–10. 10.1016/j.wsif.2013.04.007

[89] Ghanotakis E Hoke T Wilcher R Field S Mercer S Bobrow EA. Evaluation of a male engagement intervention to transform gender norms and improve family planning and hiv service uptake in kabale, Uganda. Glob Public Health. (2017) 12(10):1297–314. 10.1080/17441692.2016.1168863 27108891

[90] Shattuck D Burke H Ramirez C Succop S Costenbader B Attafuah JD. Using the inequitable gender norms scale and associated hiv risk behaviors among men at high risk for hiv in Ghana and Tanzania. Men Masc. (2013) 16(5):540–59. 10.1177/1097184(13502730

[91] R Core Team I. R: A Language and Environment for Statistical Computing. Vienna, Austria: R Foundation for Statistical Computing (2022).

[92] Cohen J. Statistical power analysis. Curr Dir Psychol Sci. (1992) 1(3):98–101. 10.1111/1467-8721.ep10768783

[93] Cohen J. Statistical Power Analysis for the Behavioural Sciences. 2nd ed. New Jersey: Lawerence Erlbaum Associates Publishers (1988).

[94] Willig C Stainton Rogers W. The Sage Handbook of Qualitative Research in Psychology. Thousand Oaks: SAGE Publishers (2025).

[95] Braun V Clarke V. Using thematic analysis in psychology. Qual Res Psychol. (2006) 3(2):77–101. 10.1191/1478088706QP063OA

[96] Jeong J Ahun MN Gunaratna NS Ambikapathi R Mapendo F Galvin L. Effects of engaging fathers and bundling parenting and nutrition interventions on early child development and maternal and paternal parenting in mara, Tanzania: a factorial cluster-randomized controlled trial. J Child Psychol Psychiatry. (2024) 65(5):694–709. 10.1111/jcpp.13897 37800367

[97] Jeong J Sullivan EF McCann JK McCoy DC Yousafzai AK. Implementation characteristics of father-inclusive interventions in low- and middle-income countries: a systematic review. Ann N Y Acad Sci. (2023) 1520(1):34–52. 10.1111/nyas.14941 36482863 PMC9974925

[98] Doyle K Bhatnagar I Karamage E Tuyisingize JP Muhimpundu C Nyiransabimana AMY. Equipping community health workers in Rwanda to deliver a gender transformative parenting program to prevent violence against women and children at scale. Front Reprod Health. (2025) 7:1602136. 10.3389/frph.2025.1602136 40625533 PMC12230087

[99] Boyer C Levy Paluck E Annan J Nevatia T Cooper J Namubiru J. Religious leaders can motivate men to cede power and reduce intimate partner violence: experimental evidence from Uganda. Proc Natl Acad Sci U S A. (2022) 119(31):e2200262119. 10.1073/pnas.2200262119 35905318 PMC9351535

[100] Siu GE Wight D Seeley J Namutebi C Sekiwunga R Zalwango F. Men’s involvement in a parenting programme to reduce child maltreatment and gender-based violence: formative evaluation in Uganda. Eur J Dev Res. (2017) 29(5):1017–37. 10.1057/s41287-017-0103-6

[101] Lachman J Wamoyi J Spreckelsen T Wight D Maganga J Gardner F. Combining parenting and economic strengthening programmes to reduce violence against children: a cluster randomised controlled trial with predominantly male caregivers in rural Tanzania. BMJ Glob Health. (2020) 5(7):e002349. 10.1136/bmjgh-2020-002349 32641291 PMC7348478

[102] Iman'ishimwe Mukamana J Machakanja P Adjei NK. Trends in prevalence and correlates of intimate partner violence against women in Zimbabwe, 2005–2015. BMC Int Health Hum Rights. (2020) 20(1):2. 10.1186/s12914-019-0220-8 31959182 PMC6971918

